# Spatial distribution of stink bugs (Hemiptera: Pentatomidae) in wheat

**DOI:** 10.1093/jis/14.1.98

**Published:** 2014-07-22

**Authors:** Francis P. F. Reay-Jones

**Keywords:** sampling, SADIE, spatial aggregation, spatial association

## Abstract

A two-year study was conducted in South Carolina wheat ( *Triticum aestivum* L. (Poales: Poaceae)) fields to describe spatial and temporal dynamics of stink bugs (Hemiptera: Pentatomidae), which were sampled weekly with sweep nets. In 2010, the main phytophagous stink bugs caught in a grid sampling plan across two fields were the brown stink bug, *Euschistus servus* (Say), the rice stink bug, *Oebalus pugnax* (F.), the southern green stink bug, *Nezara viridula* (L.), and the red shouldered stink bug, *Thyanta custator* (F.), for both adults and nymphs. In 2011, the main phytophagous stink bugs were *E. servus* , *O. pugnax* , *N. viridula* , and *T. custator* across two fields. Adult stink bug counts adjacent to fallow fields were 2.1-fold greater for all species combined compared with counts adjacent to woods. Spatial Analysis by Distance IndicEs (SADIE) indicated significant aggregation for 35% of analyses for adults and nymph stink bugs at each sampling date. As a measure of spatial and temporal stability, positive SADIE association indices among sampling dates recorded 11, 36, 43, and 16% of analyses for adult *E. servus* and 7, 50, 50, and 14% for adult *O. pugnax* in fields A, B, C, and D, respectively. Adult and nymph stink bugs were spatially associated within wheat fields based on SADIE association indices. Seasonal counts of stink bugs were spatially associated with spike counts at least once for each species across the four fields. Future work may investigate practices to reduce stink bug buildup on wheat in the spring and movement to susceptible crops such as corn, *Zea mays* L.

## Introduction

The distributions of insect populations are generally characterized by spatial heterogeneity ( Leibhold et al. 1993 ). The degree of aggregation can vary substantially between species and life stages ( Wilson 1985 ). Among methods that account for the spatial location of samples, the Spatial Analysis by Distance IndicEs (SADIE) red-blue methodology of Perry et al. (1999) can be used to identify either clusters of high-density counts or gaps of low-density counts, each in spatially nearby locations, for insect count data. The SADIE association tool can be used to describe spatial associations between two data sets that shared the same locations ( Perry and Dixon 2002 ), such as the same species taken at different times, two different species sampled together, a species and an environmental variable or crop injury expressed as counts ( Thomas et al. 2001 ; Cocu et al. 2005 ; Reay-Jones et al. 2010 ; Reay-Jones 2012 ). Understanding the spatial dynamics of insect pests of crops can assist in developing ecologically based management practices. For example, if spatial distributions are consistent among years, predictions may be used for future distributions ( Park and Tollefson 2006 ). Environmental parameters may also be used to predict pest populations ( Cocu et al. 2005 ; Díaz et al. 2010 ), which can assist in improving sampling plans or targeting control tactics.

The model insects in this study are a complex of phytophagous stink bugs (Hemiptera: Pentatomidae), which have become one of the primary pests of cotton *(Gossypium hirsutum* L.) production in the southeastern United States ( Barbour et al. 1988 ; Greene et al. 1999 , 2001). The major reason is the reduction in the amount of insecticides applied to cotton as a result of the eradication of the boll weevil, *Anthonomus grandis grandis* Bohe-man ( Haney et al. 1996 ), and the widespread adoption of transgenic cotton containing transgenes from *Bacillus thuringiensis* Berliner *(Bt)* to manage the heliothine complex ( Sullivan et al. 1996 ). Losses in U.S. cotton due to stink bug herbivory ranged in recent years from $64 million in 2005 ( Williams 2006 ) to $29 million in 2009 ( Williams 2010 ) across the cotton belt. Stink bugs can also be economic pests of other agronomic crops grown in the southeastern United States, including soybean, *Glycine max* L. Merrill ( Douce and McPherson 1991 ); corn, *Zea mays* L. ( Ni et al. 2010 ); peach, *Prunus persica* (L.) ( Porter et al. 1928 ); pecan, *Carya illinoinensis* (Wangenh.) ( Dutcher 1984 ); grain sorghum, *Sorghum bicolor* (L.) ( Young and Teetes 1977 ); tomato, *Solanum lycopersicum* L. ( Michelbacher et al. 1952 ); and wheat, *Triticum aestivum* L. (Poales: Poaceae) ( Viator et al. 1983 ).

On wheat, the southern green stink bug, *Nezara viridula* (L.), and the rice stink bug, *Oebalus pugnax* (F.), can reduce germination and kernel weight at the milk stage, but not at the dough stage ( Viator et al. 1983 ). Feeding by the Say stink bug, *Chlorochroa sayi* Stål, on wheat during the boot stage can lead to stunting and reduced kernel weight after head emergence ( Jacobson 1965 ). Insecticide applications are rarely required on wheat in the United States because insect densities are typically below economic thresholds ( McPherson and Greene 2007 ). Stink bug populations, however, may build up on wheat before migrating to more susceptible crops or other hosts for feeding and oviposition ( Jones and Sullivan 1982 ; Buntin and Greene 2004 ; Reay-Jones 2010 ). The brown stink bug, *Euschistus servus* (Say), *O. pugnax, N. viridula,* and the red shouldered stink bug, *Thyanta custator* (F.), were previously found in wheat in South Carolina ( Reay-Jones 2010 ) and Georgia ( Buntin and Greene 2004 ). Wheat is the earliest maturing agronomic crop of the year to host the buildup of stink bugs in the southeastern United States (Jones and Sullivan 1982) and can be a significant source of stink bugs that cause damage to adjacent corn ( Blinka 2008 ; Reisig 2011 ). Stink bugs colonize patches within a farm, and Ehler (2000) recommended a farmscape approach for studying and managing such mobile insects. In the southeastern United States, a farmscape can consist of patches of row crops including cotton; corn; wheat; soybeans; tobacco, *Nicotiana tabacum* L.; and peanuts, *Arachis hypogaea L.;* in addition to wooded areas and weedy field borders.

Studies on spatial distributions of stink bugs showed that *N. viridula* had a clumped pattern in soybean fields in the United States ( Todd and Herzog 1980 ) and in Japan on rice ( Hokyo and Kiritani 1962 , Nakasuji et al. 1965 ). Reay-Jones et al. (2010) also showed that stink bugs and associated boll injury were also aggregated in cotton fields in South Carolina and Georgia. A previous study on wheat showed that stink bugs were spatially aggregated based on variance-mean relationships; using inverse distance weighted interpolation and sampling transects , the study detected greater densities along the edge of wheat fields ( Reay-Jones 2010 ). This work used 10-29 sampling locations per field.

This current study aims to better characterize spatial patterns by increasing the number of sampling locations within fields, which allows the use of more comprehensive spatial methods to examine both the aggregation and association in space among species and life stages of stink bugs in wheat. The goal of this work was to provide a better characterization of the spatial patterns of stink bugs in wheat rather than identify mechanisms behind observed patterns. Because wheat has an important role in the sequence of host plants used by stink bugs during the growing season, the objective of this study was to examine the spatial aggregation of stink bugs and the spatial association between species and life stages in wheat.

## Materials and Methods

Fields and sampling procedures are identical to a previous study that reported on the spatial distribution of he cereal leaf beetle, *Oulema melanopus* (L.), in wheat ( Reay-Jones 2012 ). In 2009, wheat was planted in one field at the Pee Dee Research and Education Center in Florence, SC (field A: 7.4 ha; 34.2890 N, - 79.7384 W), and in one commercial field in Lynchburg, SC (field B: 13.6 ha; 34.0759 N, - 80.1210 W). In 010, two commercial wheat fields were planted in Lynchburg, SC (field C: 9.7 ha; 34.0877 N, -80.1145 W; field D: 9.4 ha; 34.0746 N, -80.1179 W). Conventional tillage was used in field A and no-till was used in the other three fields. Row spacing was 17.8 cm. Fields were planted with cultivar AGS 2000 (AgSouth Genetics, www.agsouthgenetics.com ) in field A on 30 November 2009 and Pioneer 21R61 (Pioneer Hi-Bred, www.pioneer.com ) in field B on 2 November 2009, and in fields C and D on 5 November 2010. No insecticides were applied in fields A and B. Lambda-cyhalothrin was applied in fields C and D at a rate of 0.028 kg (AI)/ha for *Oulema melanopus* control on 4 April 2011. The average growth stage of wheat plants was recorded at each sampling date ( Chang et al. 1974 ). The number of spikes was recorded in two 1-m row sections at each sampling location (see below) on 6 May 2010 (field A), 5 May 2010 (field B), 20 May 2011 (field C), and 5 May 2011 (field D).

### Stink bug sampling

In each field, a sampling grid consisted of one sampling location marked with a 1.8-m fiber glass pole for every 0.09 ha for field A (each pole was separated by 30 m), 0.25 ha for fields B and C (each pole was separated by 50 m), and 0.16 ha for field D (each pole was separated by 40 m). In each field, the outermost sampling location was on the edge of the field, so that sweep net sampling (see below) was conducted on the first eight rows from the field edge. Sampling locations that were located along the edge of the field were classified as “exterior” and locations within wheat fields as “interior” (see data analyses section below). Interior locations were generally located at 30 (field A), 40 (fields B and C), or 50 m (field D) from the field edge. Grid size varied with field, with 112, 81, 71, and 86 sampling locations for fields A, B, C, and D, respectively.

Although there is no minimum number of sampling locations required to use SADIE, the power of the test increases with sample size ( Perry 1998 ). A previous study on stink bug distributions in cotton showed significant SADIE aggregation indices in only 4% of species/sampling date combinations ( Reay-Jones et al. 2010 ). With a range of 15 to 28 sampling locations per field, Reay-Jones et al. suggested that an increase in sample size may help to increase the significance of spatial structure detected by SADIE. In the same fields as in the current study, 80% of adult *O. melanopus* sampling date-field combinations had significant aggregation indices ( Reay-Jones 2012 ). The grid size in the current study varied in order to have at least 70 sampling locations, while maintaining the time to sample fields manageable.

GPS coordinates of each sampling location were recorded with a Trimble GeoXM (Trimble, www.trimble.com ), and coordinates were corrected using the differential correction tool of GPS Pathfinder Office (Trimble). Stink bugs were sampled with sweep nets (38 cm diameter). At each location, two 25-pendulum sweep samples were taken across the top canopy of eight rows with each sweep. Sampling was conducted weekly from tillering to harvest (Field A: 3 March-1 June 2010, 12 sampling dates; field B: 4 March-3 June 2010, 14 sampling dates; field C: 7 March-4 May 2011, 12 sampling dates; field D: 8 March-25 May 2011, 10 sampling dates).

### Data analyses

The two 25-sweep sub-samples were summed at each sampling location for all analyses. Comparisons were made for stink bug numbers (averaged across sampling dates for each species, life stage, and across species) for locations along the edge of the field (exterior) versus sampling location within wheat fields (interior). Each field was considered a replication *(n =* 4). Average numbers of stink bugs were analyzed using an ANOVA with sampling location as fixed effect and field as random effect (PROC MIXED, SAS Institute 2011 ). Stink bug numbers also were compared for locations along the edge of woods and along the edge of fallow fields. Each field was considered a replication *(n =* 4) because woods and fallow fields bordered each field. Data were log10( *x* + 1) transformed prior to ANOVA.

Spatial Analysis by Distance IndicEs (SADIE Version 1.22, Perry et al. 1999 ) was conducted for each sampling date and year-end sums of stink bug counts. Reay-Jones (2012) reports SADIE aggregation indices for the number of wheat spikes in the same four fields, which were also spatially associated in this study with stink bug counts using the SADIE association tool (see below). The SADIE red-blue method was used for grid sampling locations expressed as absolute positions. This analysis determines the minimum distance *D* needed to achieve regularity, which is the distance moved by counts in the observed sample to reach the most uniform distribution possible. A clustering index was assigned to every location, with either a positive cluster index $({\overline v _i})$ for counts above the mean of each field-date combination or a negative gap index $({\overline v _i})$ for counts below the mean. A random spatial pattern has indices $\overline {{v_i}} = - {\overline v _i} = 1$ Non-randomness is quantified by comparing observed patterns with rearrangements in which the sample counts are randomly redistributed across the sampling locations. The overall index of dispersion *(I _a_ )* indicates either an aggregated (> 1), random (= 1), or uniform pattern (< 1). The probability *(P)* is derived after a large number of randomizations as a formal test of randomness; the null hypothesis of spatial randomness is rejected for *P <* 0.025 (aggregation) or *P >* 0.975 (uniformity) with a 5% error rate. A total of **≈** 6,000 randomizations were used for each test.

Using SADIE analyses, data sets can include counts of the same species taken at different times, two different species sampled together, or a species and an environmental variable expressed as counts ( Thomas et al. 2001 ; Cocu et al. 2005; Reay-Jones et al. 2010 ). This SADIE method was used in this study for each sampling date to determine spatial associations between sampling dates and life stages of stink bugs. The only environmental variable recorded was the number of wheat spikes. To determine whether the density of wheat spikes influenced the spatial distribution of stink bugs, the number of wheat spikes was also associated with year-end totals of each species and life stage using the SADIE association tool. An overall index of association *(X)* was determined between each of the paired datasets, with a positive association for × *>* 0 ( *P<* 0.025) and a negative association for × *<* 0 *(P* > 0.975). Mean ***X*** is calculated from the local spatial associations *(X _k_ )* at each sampling location *k.* At the local scale, a positive association between two variables indicates the presence of either a patch or a gap for both variables; a negative association indicates the presence of a patch for one variable and gap for the other variable at the same location. Interpolation maps of local aggregation indices were generated using the inverse distance weighted (IDW) spatial method with the geographical information systems software Arc View 9.2 (ESRI 2006).

### Results

In 2010, most phytophagous stink bugs caught in the grid-sampling plan across two fields included *E. servus* (43.1%, 68.8%), *O. pugnax* (40.7%, 17.8%), *N. viridula* (10.4%, 6.7%), and *T. custator* (5.7%, 6.7%), for adults and nymphs, respectively. In 2011, most phytophagous stink bugs across two fields included *E. servus* (69.3%, 88.0%), *O. pugnax* (26.7%, 9.5%), *N. viridula* (1.5%, 0.9%), and *T. custator* (2.6%, 1.6%). Other species (< 1% of total stink bugs collected) consisted of *E. tristigmus* (Say) and *E. quad-rator* (Rolston).

**Figure 1.**
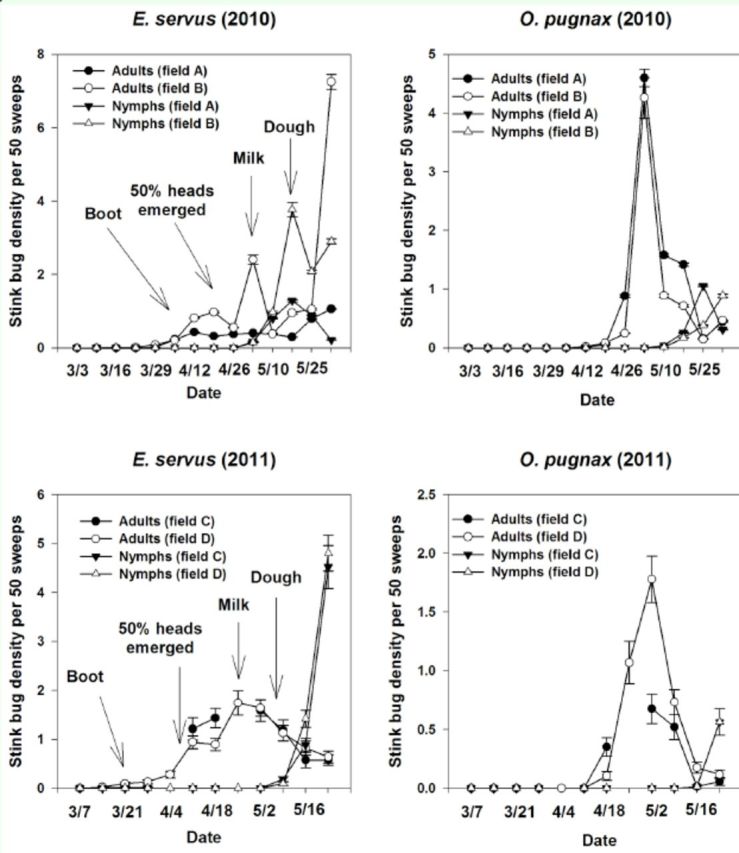
Densities of *Euschistus servus* and *Oebalus pugnax* per 50 sweeps (± SEM) in wheat fields in South Carolina in 2010 and 2011. Arrows indicate growth stage of wheat. High quality figures are available online.

**Figure 2.**
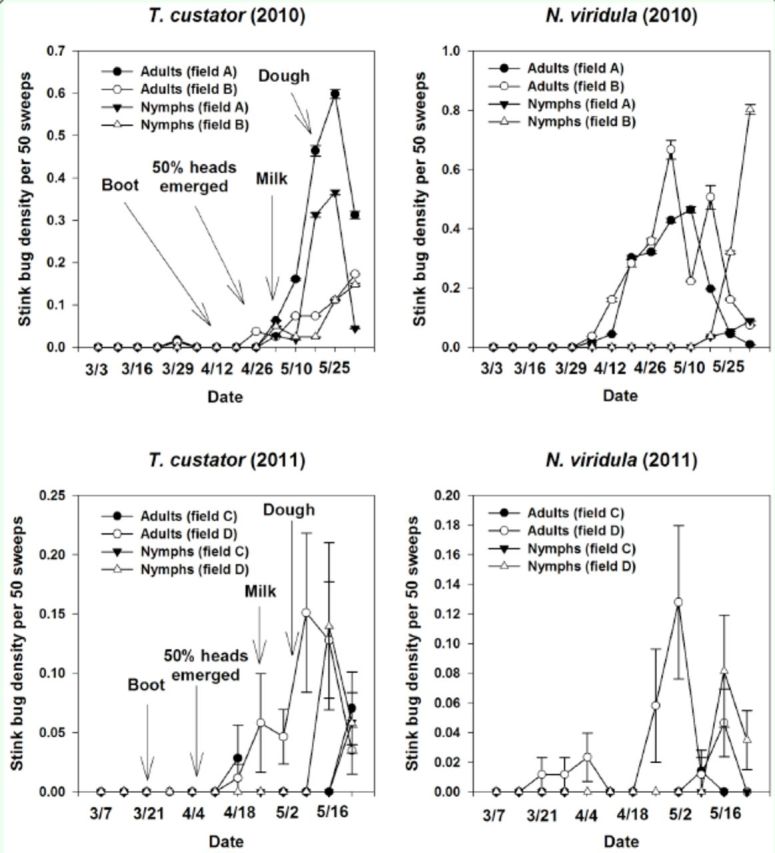
Densities of *Nezara viridula* and *Thyanta custator* per 50 sweeps (± SEM) in wheat fields in South Carolina in 2010 and 2011. Arrows indicate growth stage of wheat. Arrows indicate growth stage of wheat. High quality figures are available online.

*E. servus* adults were the first species to appear in both years during the late tillering/early boot stages ( Fig. 1 ). A population peak occurred in both years at the milk stage. Adult and nymph *E. servus* densities increased as the wheat matured. For both years, *O. pugnax* adult densities peaked at the heading stages before a decrease at early dough; this decrease coincided with an increase in nymphs ( Fig. 1 ). *T. custator* appeared at low densities at the boot stage in 2010 and at the heading stage in 2011 ( Fig. 2 ).

Densities of adult and nymph *T. custator* increased at the early dough stage. *N. viridula* appeared at the heading stage in 2010 and at the boot stage in 2011 ( Fig. 2 ). As reported in Reay-Jones (2012) , average spikes per square meter averaged 267.3 ± 7.2 (SEM) in field A, 255.6 ± 6.8 in field B, 413.9 ± 15.0 in field C, and 442.2 ± 14.6 in field D. Wheat in 2010 (field A and B) suffered from drought, which was not the case in 2011.

Stink bug abundance was not significantly ( *P* > 0.05) different among interior and exterior portions of the field for each species individually. Adult stink bugs summed across species, however, were significantly more abundant along the edge of fields (2.85 ± 0.21) than in the interior portions of fields (2.66 ± 0.22; F = 13.62; df = 1, 3; *P* = 0.0345). Trends were also observed for adult *N. viridula* (F = 6.32; df = 1, 3; *P* = 0.0867) and nymph *T. custator* (F = 7.67; df = 1, 3; *P* = 0.0696) to be 3.3-fold and 2.2-fold, respectively, more abundant along the edge of fields than in the interior portions of fields.

Stink bug abundance was not significantly ( *P* > 0.05) different adjacent to fallow fields or woods for each species individually. Adult stink bugs summed across species, however, were significantly more abundant adjacent to fallow fields (28.47 ± 5.25) than adjacent to woods (13.27 ± 3.40; F = 11.35; df = 1, 3; *P* = 0.0434). Trends also were observed for adult *N. viridula* (F = 5.80; df = 1, 3; *P* = 0.0951), adult *E. servus* (F = 5.80; df = 1, 3; *P* = 0.0775), and adult *O. pugnax* (F = 7.01; df = 1, 3; *P* = 0.0772) to be 2.9-fold, 2.2-fold, and 1.6-fold to be more abundant adjacent to fallow fields than adjacent to woods. Woods along wheat fields comprised several species; main species were loblolly pine, *Pinus taeda* L.; water oak, *Quercus nigra* L.; southern red oak, *Quercus falcata* Michaux; black cherry, *Prunus serotina* Ehrhart; and American sweetgum, *Liquidambar styraciflua* L.

According to the SADIE aggregation index, adult and nymph stink bugs were significantly ( *P* < 0.025) aggregated in field A in 13 out of 34 analyses (38%) and two out of 21 analyses (10%), respectively ( Table 1 ). In field B, adult and nymph stink bugs were significantly ( *P* < 0.025) aggregated in 21 out of 36 analyses (58%) and five out of 19 analyses (26%), respectively ( Table 1 ). In field C, adult and nymph stink bugs were significantly ( *P* < 0.025) aggregated in six out of 15 analyses (40%) and zero out of six analyses (0%), respectively ( Table 2 ). In field D, adult and nymph stink bugs were significantly ( *P* < 0.025) aggregated in eight out of 28 analyses (29%) and five out of 13 analyses (38%), respectively ( Table 2 ). Over the four fields, adults were more frequently aggregated than nymphs (42 vs. 20% of the analyses).

**Table 1.**
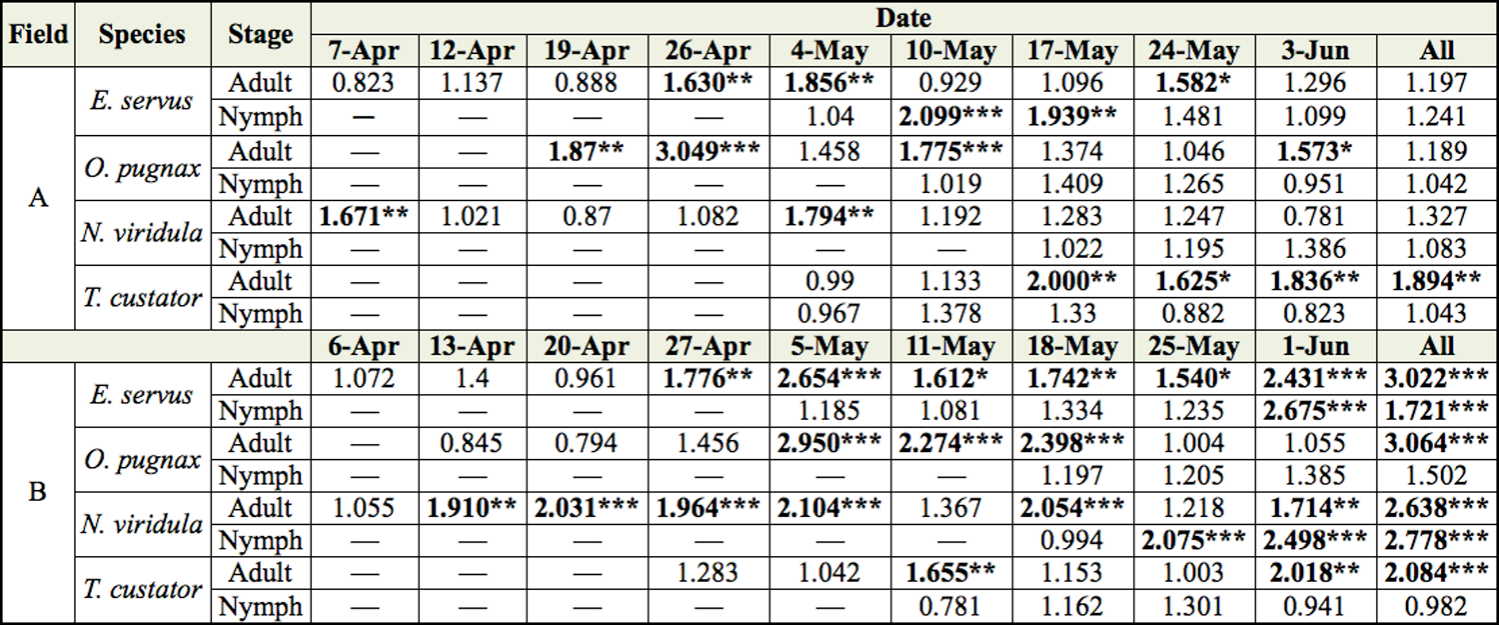
Characterization of spatial distribution of stink bug counts in fields A and B (2010) with SADIE index of clustering ( *Ia* ). *Ia* = 1 suggests a random, *Ia* =1 suggests an aggregated, and *Ia* 1 suggests a regular spatial patterns. Significant ( *P* < 0.025) aggregations are in bold.

*** *P* < 0.001; ** *P* < 0.01; * *P* < 0.025

**Table 2.**
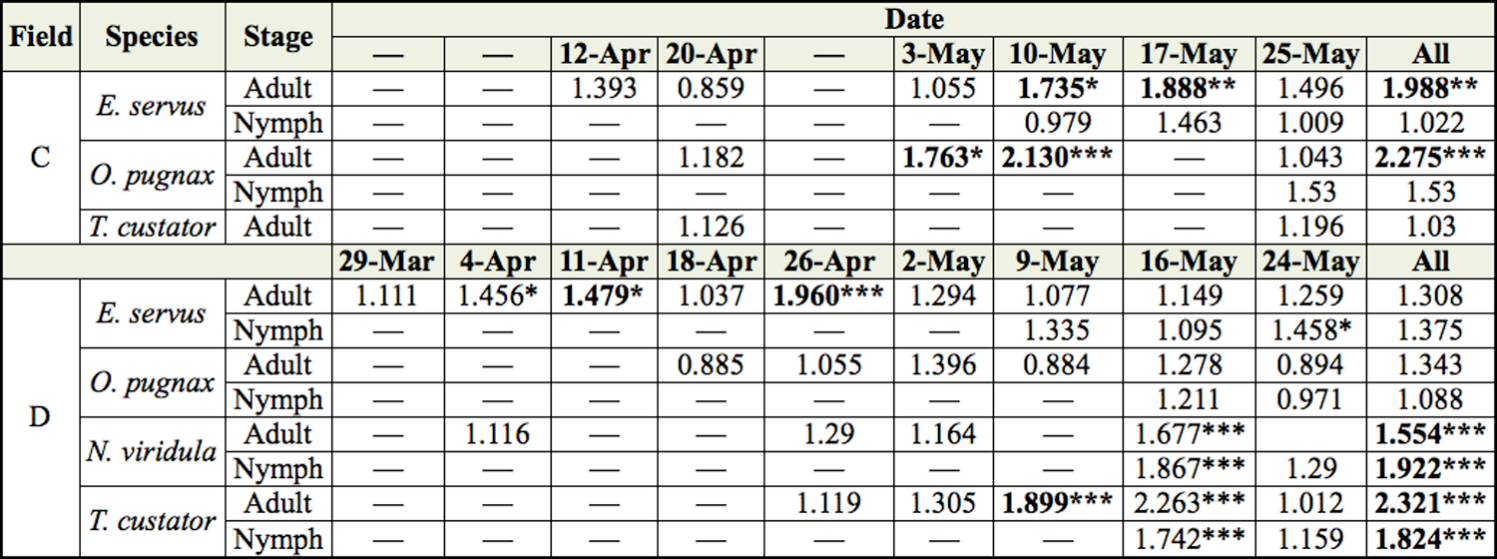
Characterization of spatial distribution of stink bug counts in fields C and D (201 1) with SADIE index of clustering ( *I _a_* ). *I _a_* = 1 suggests a random, *I _a_ >* 1 suggests an aggregated, and *I _a_* 1 suggests a regular spatial patterns. Significant ( *P* < 0.025) aggregations are in bold.

*** *P* <0.001; ** *P* < 0.01; * *P* < 0.025. Numbers of *Nezara viridula* adults and nymphs, and *Thyanta custator* nymphs were insufficient to run SADIE analysis in field C. **v.**

**Figure 3.**
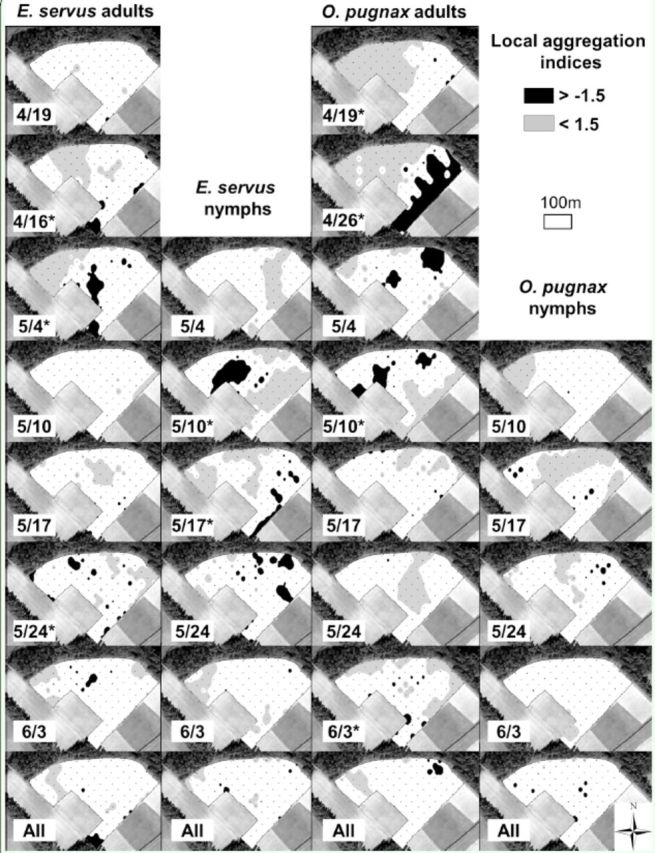
Spatial interpolation of SADIE gaps and clusters of stink bug local aggregation indices in field A, Florence County, SC, 2010. Missing dates indicate that insect counts were insufficient to generate local aggregation indices. Asterisks next to dates indicate significant ( *P* < 0.025) aggregations. High quality figures are available online.

**Figure 4.**
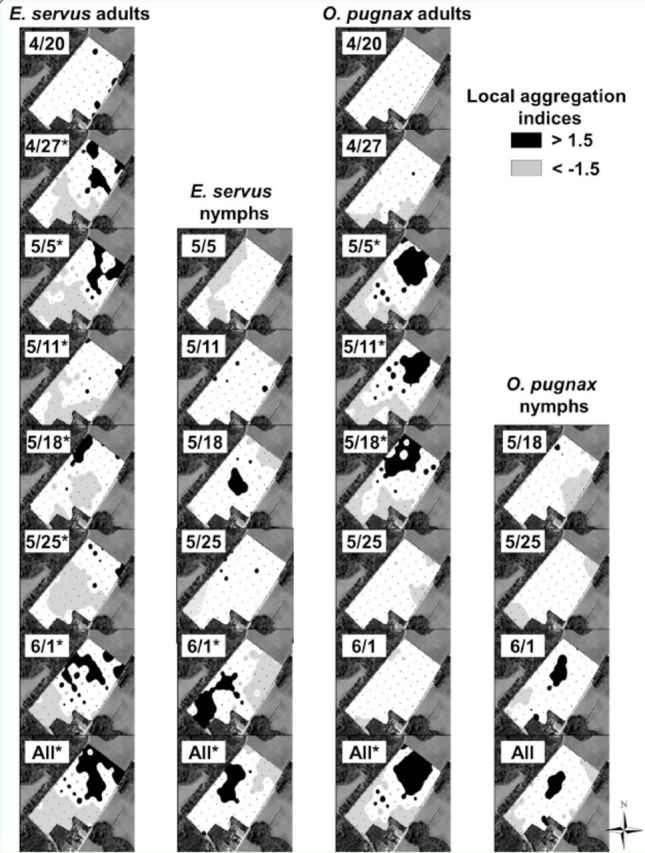
Spatial interpolation of SADIE gaps and clusters of stink bug local aggregation indices in field B, Lee County, SC, 2010. Missing dates indicate that insect counts were insufficient to generate local aggregation indices. Asterisks next to dates indicate significant ( *P* < 0.025) aggregations. High quality figures are available online.

**Figure 5.**
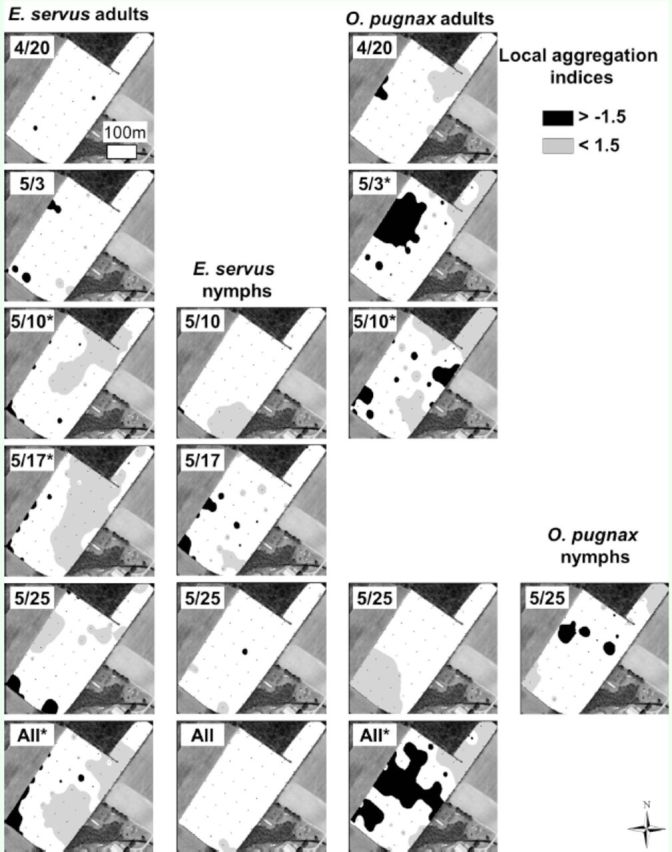
Spatial interpolation of SADIE gaps and clusters of stink bug local aggregation indices in field C, Lee County, S.C, 2011. Missing dates indicate that insect counts were insufficient to generate local aggregation indices. Asterisks next to dates indicate significant *(P* < 0.025) aggregations. High quality figures are available online.

**Figure 6.**
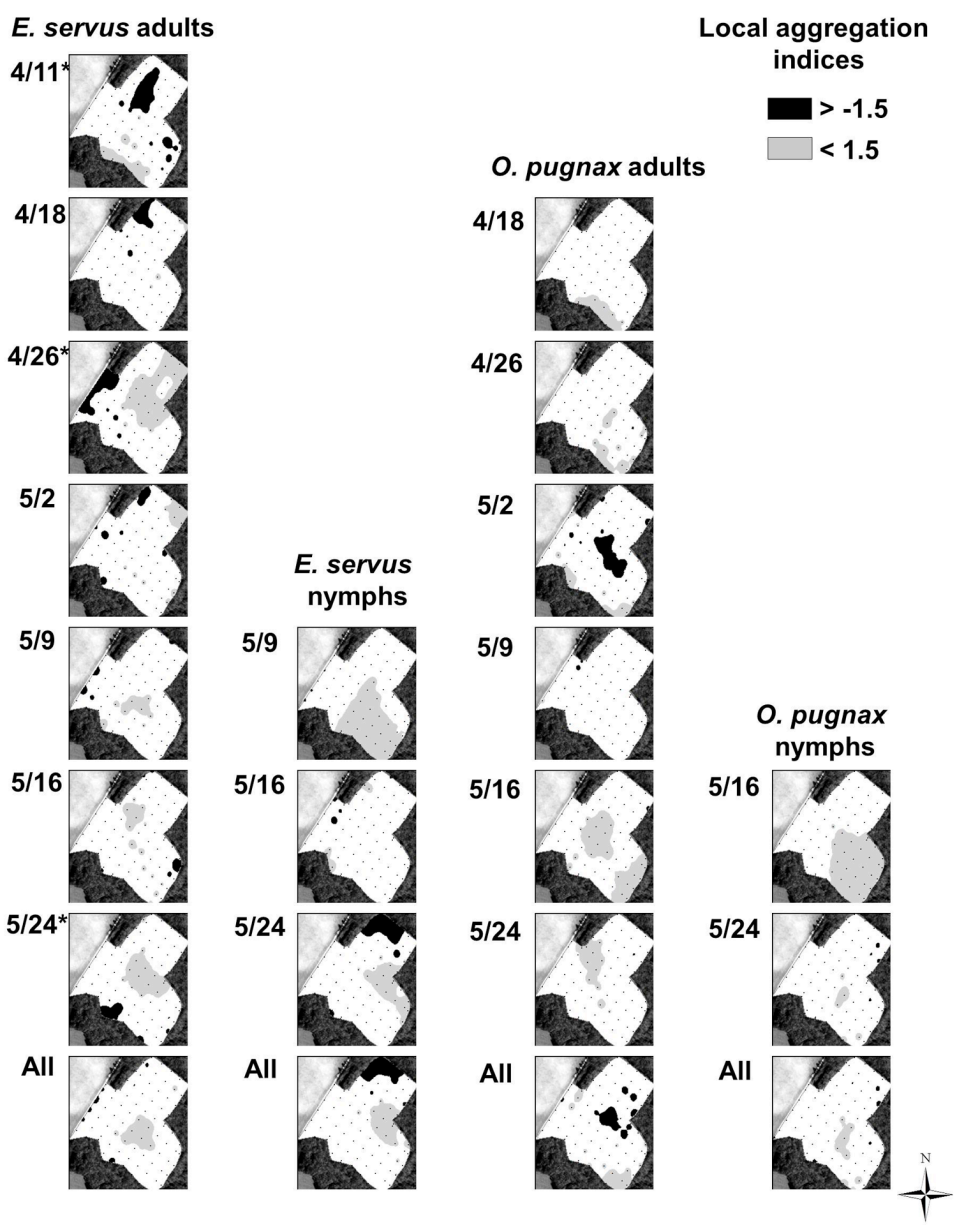
Spatial interpolation of SADIE gaps and clusters of stink bug local aggregation indices in field D, Lee County, SC, 2011. Missing dates indicate that insect counts were insufficient to generate local aggregation indices. Asterisks next to dates indicate significant *(P* < 0.025) aggregations. High quality figures are available online.

Local aggregation indices, shown only for adults of the two most abundant species ( *E. servus* and *O. pugnax* ), showed variability between dates ( Fig. 3 ‒6). Spatial and temporal stability, as indicated by positive association indices among sampling dates, were significant in 11, 36, 43, and 16% of analyses for adult *E. servus* and 7, 50, 50, and 14% for adult *O. pugnax* in fields A, B, C, and D, respectively ( Tables 3 and 4 ). For example, significant associations are readily apparent for *O. pugnax* in field B between 5, 11, and 18 May sampling dates, with significant gaps and patches generally in similar locations within the field. Similarly, the significant positive association for *E. servus* adults between 27 April and 5 May was also apparent ( Fig. 4 ).

**Table 3.**
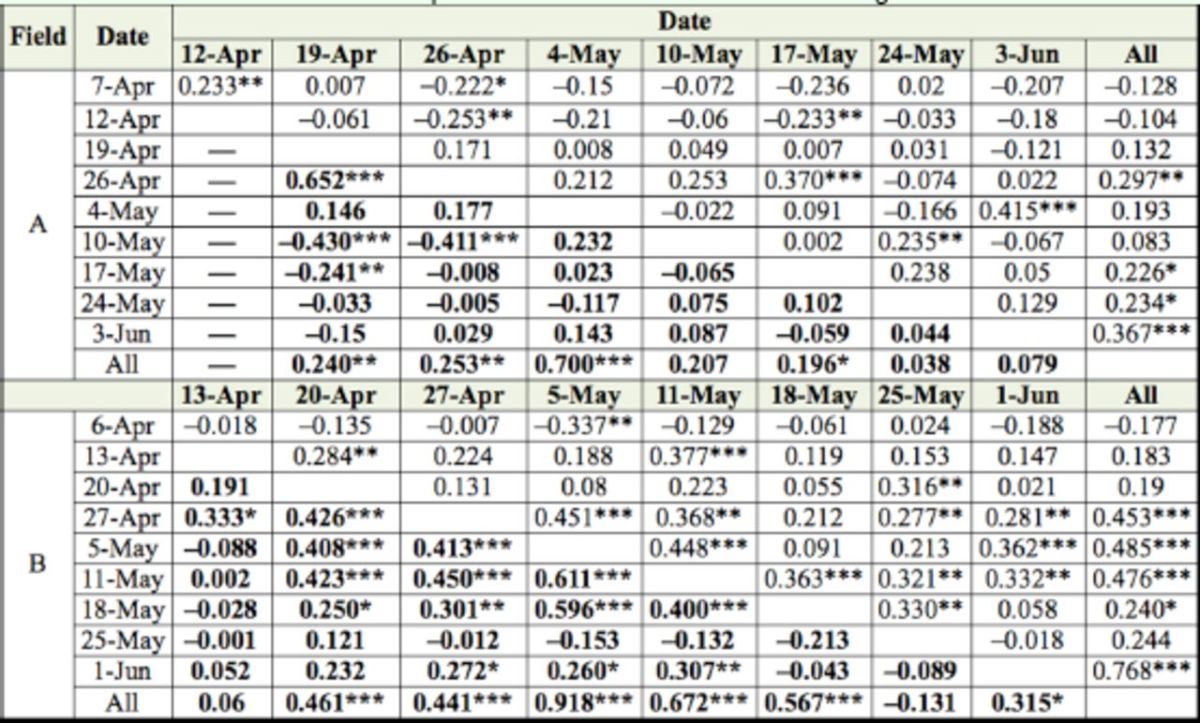
Intraspecific association analyses between sampling dates for *Euschistus servus* and *Oebalus pugnax* (bold font) densities in 2010. Numbers are association indices with a positive association for *P* < 0.025 and a negative association for *P >* 0.975.

*** *P* < 0.001; ** *P* < 0.01; * *P* < 0.025

**Table 4.**
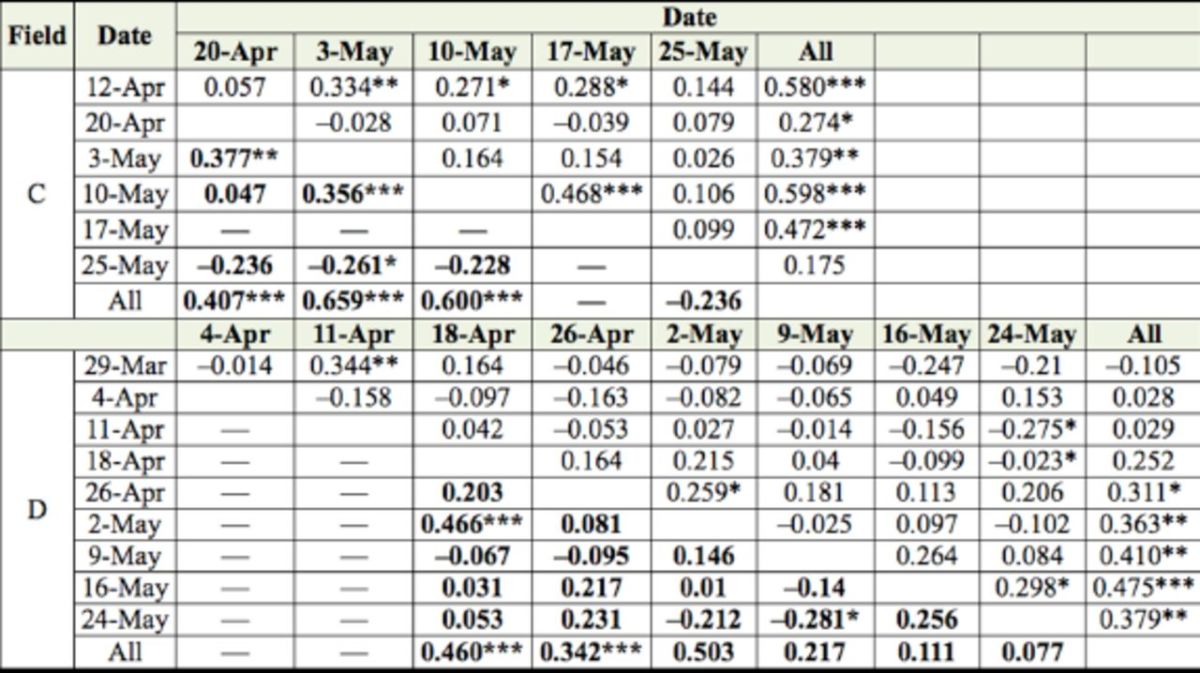
Intraspecific association analyses between sampling dates for *Euschistus servus* and *Oebalus pugnax* (bold font) densities in 201 1. Numbers are association indices with a positive association for *P* < 0.025 and a negative association for *P >* 0.975.

*** *P <* 0.001; ** *P* < 0.01; * *P* < 0.025.

For field A, significant positive spatial associations were detected between *E. servus* adults on 4 May and nymphs on 17 May ( *Χ* = 0.476, *P* < 0.0001), 24 May ( *Χ* = 0.197, *P* = 0.024), and 3 June (× *=* 0.273, *P =* 0.0006). For *O. pugnax* in the same field, significant positive spatial associations were detected between adults on 19 April and nymphs on 10 May ( ***X*** = 0.334, *P =* 0.001) and 24 May *(X=* 0.260, *P =* 0.008), adults on 26 April and nymphs on 10 May *(X=* 0.283, *P =* 0.017) and 24 May ( ***X*** = 0.319, *P =* 0.003), adults on 10 May and nymphs on 3 June ( ***X*** = 0.233, *P =* 0.016), adults on 17 May and nymphs on 17 May (× *=* 0.224, *P =* 0.018), adults on 3 June and nymphs on 10 May (× *=* 0.191, *P =* 0.034). For field B, significant positive spatial associations were detected between *E. servus* adults on 18 May and nymphs on 1 June *(X=* 0.286, *P =* 0.007) and *O. pugnax* adults on 25 May and nymphs on 1 June ( ***X*** = 0.402, *P =* 0.0002). For field C, significant positive spatial associations were detected between *E. servus* adults on 10 May and nymphs on 17 May (× *=* 0.400, *P =* 0.006). Nymphs were not spatially associated with adults in field D.

Total numbers of stink bugs were not associated with wheat spike numbers in field A *(P >* 0.05) ( Table 5 ). In field B, spike numbers were spatially associated with *N. viridula* and nymphs, *E. servus* adults, *T. custator* adults, and *O. pugnax* adults and nymphs. In field C, only *O. pugnax* adults were associated with wheat spike numbers. In field D, *O. pugnax* adults and *N. viridula* nymphs were associated with wheat spike numbers.

**Table 5.**
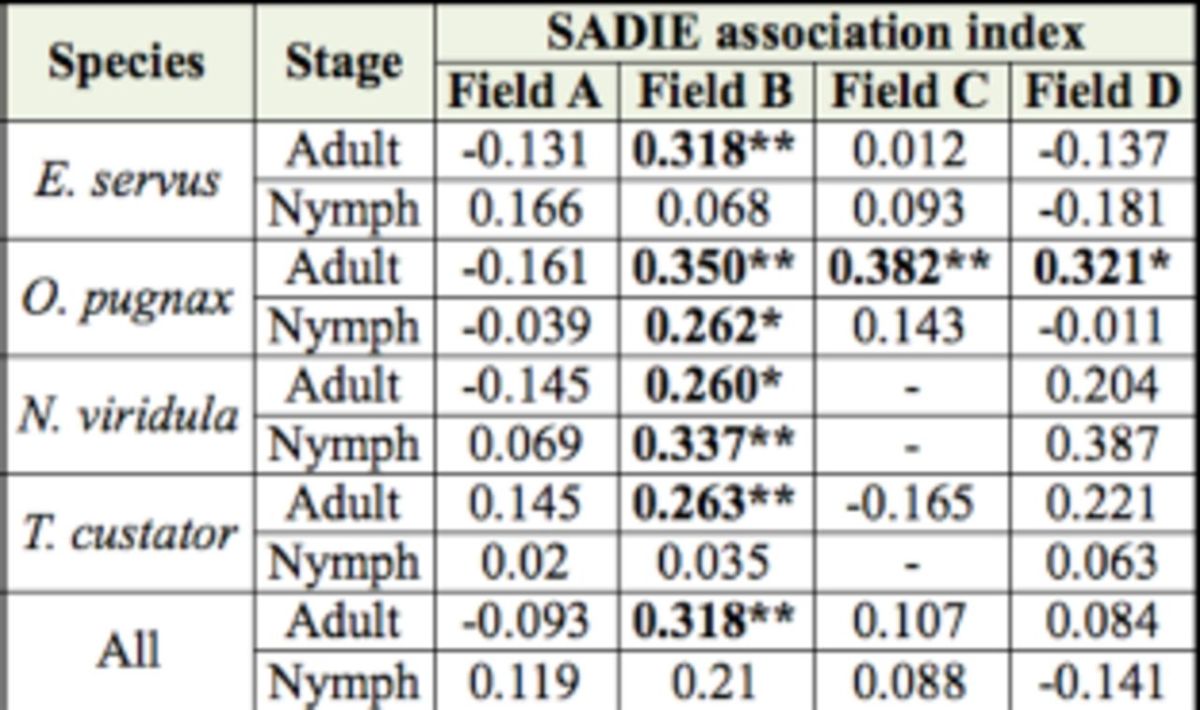
Association analyses between seasonal numbers of stink bugs and wheat spike numbers. Numbers are association indices with a positive association for *P* < 0.025 and a negative association for *P x003E;* 0.975.

*** *P <* 0.001; ** *P* < 0.01; * *P* < **I** 0.025

### Discussion

Averaging over locations at the interior vs. the edge of the field, this study showed greater densities across sampling dates along the edge of fields only for all species combined, with strong trends for *N. viridula* adults and *T. custator* nymphs. A previous study ( Reay-Jones 2010 ) in wheat in South Carolina showed that densities of stink bugs along the edge of wheat fields were greater at 0 and 5 m from the edge of the field than at 10 and 25 m. Because the grid size varied from 30 to 50 m, many of the flags along the edge of the fields were at least 10 m distant from flags that were classified as being in the interior portions of the field. A previous study ( Reay-Jones 2012 ) on spatial patterns of the *Oulema melanopus* from the same wheat fields sampled in this study reported significantly higher densities of wheat spikes along the edge of fields compared with interior portions of the field and along fallow fields compared with woods. The shading of wheat by trees surrounding fields in some places likely slowed the growth of wheat compared with interior portions of the field, which may have reduced the likelihood of a stronger edge effect for stink bug densities. As a likely effect of shading, the number of wheat spikes was significantly greater in interior vs. exterior portions of the fields, indicating that stink bugs may have favored interior portions of the fields for feeding. A similar pattern was observed in these fields for *O. melanopus.* Spike numbers also were significantly greater adjacent to fallow fields than adjacent to woods ( Reay-Jones 2012 ), again likely because of shading along the woods that hindered wheat development. Greater densities of stink bugs occurred along the edge of fallow fields for all species combined only, with strong trends for *N. viridula, E. servus,* and *O. pugnax* adults. Edge effects can be caused by the plant structure of crops, which acts as barriers to dispersal (Tillman et al. 2010); fallow fields may therefore serve as a corridor of movement of stink bugs into wheat. Future work to identify mechanisms behind observed distributions of stink bugs in wheat should consider the effect of resource concentration on insect dynamics.

Edge effects have been shown for stink bugs on other crops. On cotton, a significant decrease in both stink bug densities and associated boll injury in cotton occurred from 0 to 25 m into the field ( Reeves et al. 2010 ). Greater densities of stink bugs along the edge of fields have also been reported for *O. pugnax* in rice ( Espino et al. 2008 ), *Euschistus conspersus* Uhler in sugar beets *(Beta vulgaris* L), cotton, and sorghum ( Toscano and Stern 1976 ), and *E. conspersus, Thyanta pallidovi-rens* (Stål), *N. viridula* and *Chlorochroa uhleri* (Stål) in tomatoes ( Zalom et al. 1996 ), and *N. viridula* and *E. servus* along a cotton-peanut interface ( Tillman et al. 2009 ). Such edge effects could potentially be exploited by localized insecticide applications in susceptible crops such as cotton ( Reay-Jones et al. 2010 ). Though densities rarely reach economic thresholds in wheat, migration to adjacent corn fields as wheat matures can sometimes warrant the need for control measures in corn ( Reisig 2011 ).

35% of all SADIE indices of aggregation for adults and nymph stink bugs indicated significant aggregation at **α***=* 0.05 level in this study. This was considerably more than reported in a previous study on cotton where stink bug densities summed across sampling dates had significant aggregation indices for only 4% of analyses, in part because of low densities of some of the species and life stages in several fields ( Reay-Jones et al. 2010 ). Low densities can reduce the sensitivity of SADIE ( Thomas et al. 2001 ). The distance to regularity *D* used for the SADIE *I _a_* index is influenced by the relative and absolute position of clusters ( Perry and Klukowski 1997 ). The *I _a_* index is influenced more by the number and position of the clusters than cluster size ( Xu and Madden 2004 ). The number of possible clusters and gaps within a field increases with the number of sampling locations. An average of 87 sampling locations was used in this study, compared with 21 used in Reay-Jones et al. (2010) . This increase in the size of the field and the number of sampling locations might have increased the significance of spatial structures detected by SADIE.

Clusters of stink bugs along the edge of wheat fields were apparent in some cases in maps of local aggregation indices (for example, *E. servus* adults in the southeastern edge of field C on 17 and 25 May). Clusters are defined as several nearby large counts ( Perry et al. 1999 ). If densities do drop off rapidly after 5 m along the edge of wheat fields ( Reay-Jones 2010 ), the resolution of the grid may not have been fine enough to have several nearby large counts necessary to detect edge effects. Edge effects and differences among spatial scales have previously been shown to affect the observed spatial patterns of aphids ( Winder et al. 1999 ) and stink bugs in wheat ( Reay-Jones 2010 ). Corn is also the only major host crop available to stink bugs in late May-early June when wheat is typically harvested in the southeastern United States. No corn crop was immediately adjacent to wheat fields in this study. By mid-May, surrounding fields were either fallow or newly planted cotton was not yet attractive to stink bugs. The lack of an interface between two host crops, where stink bugs can build up ( Tillman et al. 2009 , Reay-Jones et al. 2010 , Reeves et al. 2010 ) also may have reduced detectable edge effects.

Spatial and temporal stability, as indicated by positive association indices among sampling dates, varied from 11 to 43% for adult *E. servus* and 7 to 50% for adult *O. pugnax* across the four fields. These similarities in local aggregation indices across sampling dates as detected by association indices are apparent in some fields. For instance, higher densities of *E. servus* adults were apparent in field B along the north eastern side of the field on 27 April, 5 May, 11 May, 25 May, and 1 June. Gaps and clusters of *E. servus* were also spatially associated between 10 and 17 May and clearly visible in field C. Such stability in the spatial distributions of stink bugs within fields is not necessarily evident, as *N. viridula* can disperse up to 1,000 m per day in search of sites in which to feed and oviposit ( Kiritani and Sasaba 1969 ). Thomas et al. (2001) showed that accumulated totals of carabid beetles summed across sampling dates in a hedgerow and two arable fields had significant SADIE aggregation indices. Despite changes in the location of clusters from week to week, the patch structure clearly persisted sufficiently to yield significant SADIE aggregation indices. Accumulated totals of stink bugs in this study were significantly aggregated in 13 of 29 analyses. This stability likely indicates that stink bug species were responding consistently to environmental factors The only environmental parameter recorded in this study was the number of wheat spikes per square meter at each sampling location, which were previously reported to be aggregated in two fields (B and D) and randomly distributed in two fields (A and C) ( Reay-Jones 2012 ). Seasonal counts of stink bugs were associated with spike counts at least once for each species across the four fields. Positive associations indicated that higher stink bug densities sometimes coincided with areas where spike counts were higher. Because stink bugs feed on developing wheat kernels (Jacobson 1965, Viator et al. 1983 ), it is likely that stink bugs are able to avoid areas in the field where resources are scarce.

Except for a few *N. viridula* individuals in 2011, *E. servus* was the first species to build up on wheat in both years, with adults appearing at the late boot/early heading stages. *O. pugnax* and *N. viridula* densities peaked at the milk/dough stage in both years and decreased substantially by harvest. *T. custator* began in creasing at the early milk stage. Nymph densities of all four species increased as the wheat head matured. Small grain crops serve as a major source of first-generation stink bugs in the southeast United States ( Jones and Sullivan 1982 , Todd 1989 , Reay-Jones 2010 ). As in Reay-Jones (2010) , adult stink bugs in this study began to build up on wheat from the boot stage onward; these adults likely emerged from overwintering and migrated to wheat fields. Leaf litter under deciduous trees or pine trees found in our study around wheat fields have previously been shown to serve as overwintering sites for *E. servus, O. pugnax,* and *N. viridula* ( Jones and Sullivan 1981 ). Similar observations are found in rice where stink bugs first appear once heads are available ( Douglas 1939 , Jones and Cherry 1986 ). Density peaks of stink bugs generally coincide with or lag behind the development of reproductive stages of major host plants ( Todd 1989 ).

Control measures are likely not warranted on wheat based on the densities measured in this study, as was the case in previous studies on wheat in Georgia ( Buntin and Greene 2004 ) and South Carolina ( Reay-Jones 2010 ). Viator et al. (1983) showed that densities of two stink bugs (either *N. viridula* or *O. pugnax)* per 20 spikes damaged wheat at the milk and dough stages in greenhouse studies. A maximum of 5.0 and 1.9 *N. viridula* or *O. pugnax* bugs per 50 sweeps was found at the heading stage in this study in 2010 and 2011, respectively. This corresponds to 0.037 and 0.086 stink bug per 20 spikes, therefore well below the threshold of two. In fields C and D, an application of a pyrethroid insecticide was made in early April for *Oulema melanopus* control. Before this date, a limited number of *E. servus* and *N. viridula* were found in field D. *E, servus,* the most abundant species at the time of application, is more tolerant to pyrethroids than *N.*

*viridula* (Greene et al. 2001, Willrich et al. 2003 ). In addition, stink bug densities generally begin to peak after mid-April in South Carolina when heads are available, as seen in field A and B and in Reay-Jones (2010) . It is likely that the insecticide application had a limited impact on stink bug populations only during the two weeks after application, and it did not prevent stink bugs from invading fields C and D by mid- to late April.

In addition to confirming findings of a previous study showing that stink bugs were aggregated in wheat, the use of SADIE in this study shows the temporal and spatial association among life stages and species of stink bugs. Although it was not my purpose to identify causal factors affecting aggregation, this work suggests that wheat head densities influence the buildup of stink bugs. Because stink bug dispersal to corn when wheat senesces can be an economic problem in the southeastern United States ( Blinka 2008 , Reisig 2011 ), management practices to mitigate this issue may investigate cultural practices that affect stand count and phenology. Determining the dynamics of stink bugs in farmscapes can provide useful information that could lead to localized insecticide applications or environmentally friendly control tactics such as trap cropping or other practices to enhance naturally occurring parasitoids or predators. This study showed inconsistent edge effects, despite earlier work showing greater densities along the edge of wheat ( Reay-Jones 2010 ) and cotton fields in South Carolina ( Reay-Jones et al. 2010 , Reeves et al. 2010 ). This is the first work to quantify the spatial association among life stages and species of stink bugs in wheat. More permanent and sustainable solutions to insect pest problems can result from using ecology-based control methods. The development of our understanding of the ecology of key pests such as stink bugs may help to identify strategies that will mitigate their effect on major field crops and provide environmentally friendly strategies.
